# Royal College of Surgeons—Mr. Guthrie's Lectures

**Published:** 1830-07-01

**Authors:** 


ROYAL COLLEGE OF SURGEONS.

MR. GUTHRIE completed his Surgical Lectures at the College on Saturday, the 15th instant. Having noticed the injuries of the head, of the chest, and the diseases of the urethra; on the last day, he shewed the instrument used for breaking a stone in the bladder, with an improvement which, he said, was of the utmost importance, rendering it a perfectly safe instrument, which it was not before. It could now be taken to pieces in the bladder, and each piece might be drawn out separately, so that no accident could now arise, capable of preventing its withdrawal. He stated that he was led to make this improvement, with the assistance of Mr. Weiss, (to whose ingenuity he was much indebted), in consequence of having used the instrument near five years ago, on which occasion it became clogged and could not for a long time be cleared so as to be withdrawn. The patient would not admit of its re-introduction, and was cured by the common operation, which he performed a week afterwards. He had been assured that in two instances in France an accident of this kind had happened;—the instrument could not be withdrawn, and the patients died. He therefore considered an improvement which obviated this difficulty as one of the greatest importance, as regarded the safe application of the instrument.

